# Head posture before and after the correction of unilateral functional posterior crossbite in growing children: a prospective controlled clinical trial

**DOI:** 10.1093/ejo/cjaf096

**Published:** 2025-12-18

**Authors:** Mustafa Al-Yassary, Kelly Billiaert, Fara Beltrami, Stavros Kiliaridis, Gregory S Antonarakis

**Affiliations:** Division of Orthodontics, University Clinics of Dental Medicine (CUMD), Faculty of Medicine, University of Geneva, rue Michel-Servet 1, 1206 Geneva, Switzerland; Division of Orthodontics, University Clinics of Dental Medicine (CUMD), Faculty of Medicine, University of Geneva, rue Michel-Servet 1, 1206 Geneva, Switzerland; Division of Orthodontics, University Clinics of Dental Medicine (CUMD), Faculty of Medicine, University of Geneva, rue Michel-Servet 1, 1206 Geneva, Switzerland; Division of Orthodontics, University Clinics of Dental Medicine (CUMD), Faculty of Medicine, University of Geneva, rue Michel-Servet 1, 1206 Geneva, Switzerland; Department of Orthodontics and Dentofacial Orthopedics, Dental School/Medical Faculty, University of Bern, Hochschlstrasse 4, 3012 Bern, Switzerland; Division of Orthodontics, University Clinics of Dental Medicine (CUMD), Faculty of Medicine, University of Geneva, rue Michel-Servet 1, 1206 Geneva, Switzerland

**Keywords:** head posture, functional posterior crossbite, inertial measurement unit, prospective controlled clinical trial

## Abstract

**Objective:**

To evaluate the impact of unilateral functional posterior crossbite, as well as its correction, on head posture in growing children in comparison to children without crossbite.

**Methods:**

A prospective longitudinal controlled clinical trial was carried out including 40 children aged 8–11 years, assigned into a treatment group with unilateral functional posterior crossbite treated with maxillary expansion (*n* = 20) and an untreated control group without malocclusion (*n* = 20). Head posture was assessed pre-treatment (or pre-follow-up) (T0) and 2.5 years post-treatment (or post-follow-up) (T1) using an inertial measurement unit (IMU) measuring pitch (forward/backward head inclination), roll (left/right tilting), and yaw (left/right head rotation). Dominant-eye determination was performed using the Battista Della Porta aiming test to evaluate its potential influence on head posture.

**Results:**

No significant differences in head posture were observed between the crossbite and control groups pre- (T0) or post-treatment (T1). Within the crossbite group, an increased pitch post-treatment was noted (*P* = .008), although not significant after Bonferroni correction. No significant changes were found in the roll or yaw axes neither between groups nor across time points. Eye dominance influenced yaw orientation, with right-eye-dominant participants showing higher yaw angles compared to left-eye-dominant participants (3.61+/−3.46° versus −1.47+/− 3.46°; *P* < .001).

**Conclusion:**

The presence of a unilateral functional posterior crossbite does not appear to influence head posture, when compared with a control group without crossbite. Furthermore, the orthodontic correction of unilateral functional posterior crossbite does not seem to alter head posture. The results question a cause–effect relationship between crossbite and head posture. The study did identify however a significant influence of eye dominance on head posture.

## Introduction

It has previously been found that individuals with untreated unilateral functional posterior crossbite have thinner masseter muscles on the crossbite side compared with the normal side possibly due to asymmetric muscle activity [1]. Orthodontic correction of this malocclusion may lead not only to an improved dental occlusion but also to a restored functional symmetry of the masticatory muscles and hence masticatory function.

Unilateral functional posterior crossbite, often linked to asymmetrical masticatory muscle activity, may influence maxillomandibular growth and result in adaptive skeletal and postural changes [2]. Prior research suggests that posterior crossbite may alter head posture through mandibular functional shifts, potentially affecting cervical alignment or spinal flexion [3–5].

Natural head posture (NHP) is a fundamental concept in oral rehabilitation and orthodontics and is defined as the most balanced head position when a person’s visual axis is horizontal, either standing or sitting [6].

Previous studies have explored the relationship between malocclusions, such as posterior crossbite, and pronounced postural deviations, including scoliosis. A review of the literature concluded that there is plausible evidence for an increased prevalence of unilateral Angle Class II malocclusions associated with scoliosis and an increased risk of posterior crossbite in children affected by scoliosis [7]. Another study found that individuals with unilateral posterior crossbite exhibited significant differences in horizontal alignment at the acromion and anterior-superior iliac spine levels compared with controls, suggesting that posterior crossbite is related to static body posture [8]. These findings indicate a potential association between dental malocclusions and more pronounced postural alterations, such as scoliosis, highlighting the importance of interdisciplinary approaches in diagnosis and treatment.

This cause–effect relationship between posture and malocclusions such as posterior crossbite however remains debated. Limited evidence supports a consistent relationship between posterior crossbites and long-term postural adaptations [9]. Some studies indicate that head posture and craniofacial morphology may remain unaffected by the presence of a crossbite, with individual growth patterns and neuromuscular adaptability playing a role [3, 10–12].

The potential for head posture improvement after crossbite correction also remains inconclusive. While some studies report that orthodontic treatment could have some benefits, the variability in methodologies and sample sizes affects reliability [2]. Other authors find minimal or inconsistent effects regarding the benefit of orthodontic treatment in the improvement of head posture [9]. Moreover, most existing studies use one-shot and two-dimensional methods to evaluate head posture, lack standardization, or provide subjective assessments of head posture without quantifiable measurements. These may present important limitations since head posture is both dynamic (shows variation over time) and a three-dimensional position.

The present study thus aims to: (i) evaluate the impact of unilateral functional posterior crossbite on dynamic three-dimensional head posture in growing children in comparison to children without crossbite; (ii) evaluate the impact of orthodontic correction of unilateral functional posterior crossbite on dynamic three-dimensional head posture in growing children.

The null hypotheses were that: (i) there are no differences in head posture between children with unilateral functional posterior crossbite and children without crossbite; and (ii) there are no differences in head posture before and after the orthodontic correction of unilateral functional posterior crossbite in growing children.

## Materials and methods

### Study design

This present prospective longitudinal controlled clinical trial was approved by the local ethics committee (IRB approval number CCER 2020-01227) and conducted in accordance with the Declaration of Helsinki. The reporting of this study adhered to the Strengthening the Reporting of Observational Studies in Epidemiology (STROBE) guidelines [13]. The sample consisted of two groups: a treatment group of children with unilateral functional posterior crossbite treated with maxillary expansion, and a control group without transverse malocclusions that did not receive orthodontic treatment. Head posture measurements were taken at two different time points, prior to treatment (T0) and 2.5 years after the correction of the posterior crossbite including the retention period (T1), with the expansion appliance kept in place passively for retention for 9 months following expansion. The equivalent time frame was used in the untreated control group for the T0 and T1 measurements.

### Participants, eligibility, and setting

Participants were recruited from the Division of Orthodontics at the University Clinics of Dental Medicine, University of Geneva, Switzerland, between September 2020 and October 2021. Both the patients and their parents were informed about the study procedures, and written informed consent was obtained prior to participation. The target sample size was 40 patients, with 20 patients in the treatment group and 20 in the control group. The sample size calculation was based on the primary outcome of the present clinical trial, which was to compare masseter muscle thickness in individuals with and without functional posterior crossbite [14].

Eligibility criteria for the treatment group were as follows: patients aged 8–11 years, in the mixed dentition stage, with a unilateral functional posterior crossbite accompanied by a mandibular shift towards the crossbite side. Patients were excluded if they were outside the age range, had a crossbite without a mandibular shift or had a bilateral crossbite, had undergone previous orthodontic treatment or with the presence of a space maintainer, had craniofacial syndromes, clefts, temporomandibular disorders, were medically compromised, had muscular or neuromuscular disorders, or if they were unwilling to participate. The control group met the same criteria as the treatment group, except that they had no unilateral posterior crossbite or any other transverse malocclusion of dental or skeletal asymmetry and had no immediate need for orthodontic treatment.

### Intervention (treatment and control groups)

In the treatment group, unilateral posterior crossbites were corrected using either a quadhelix or a Hyrax-type expander. The Hyrax expander was activated with one-quarter turn per day (0.25 mm) [15], while the quadhelix expander was activated one molar width, which corresponds to ∼3–4 mm per side at every appointment, until the palatal cusps of the upper first molars contacted the buccal cusps of the lower first molars. Over-expansion was prescribed for all patients, with the same transverse endpoint reached. Expansion appliances were retained passively for 9 months after expansion for transverse retention [16], and the patients were subsequently followed up until 2.5 years after expansion. The specific appliance type was not considered a critical variable for the study outcome, as both the quadhelix and Hyrax expanders achieve similar correction of the unilateral posterior crossbite. The main objective was the resolution of the functional shift and the establishment of normal transverse relationships. The selection between appliances depended primarily on the clinician’s preference and patient-specific factors. The control group was to receive no orthodontic treatment during the study period.

### Experimental protocol

Natural head posture (NHP) measurements were obtained on two separate occasions, the first recording before the start of the orthodontic treatment (T0), and the second recording 30 months after the correction of the unilateral functional posterior crossbite (or an equivalent time period in the control group) (T1). The protocol for measuring NHP was adapted from a previous study [17, 18] and was carried out as detailed below.

The participants sat comfortably in a chair with a standardized 90–90–90 position (90° angle at the hips, knees, and feet) [19] to minimize any body posture influences on head position. Participants were instructed to look into their own eyes in a mirror for 30 seconds to establish a baseline head posture (mirror-guided head posture). This step ensures that the participants achieve a consistent starting point for the subsequent self-guided natural head posture. The mirror was then covered up, and participants were asked to maintain their head in a balanced position for 5 minutes without the aid of a mirror (self-guided natural head posture) [17].

**Figure 1.**
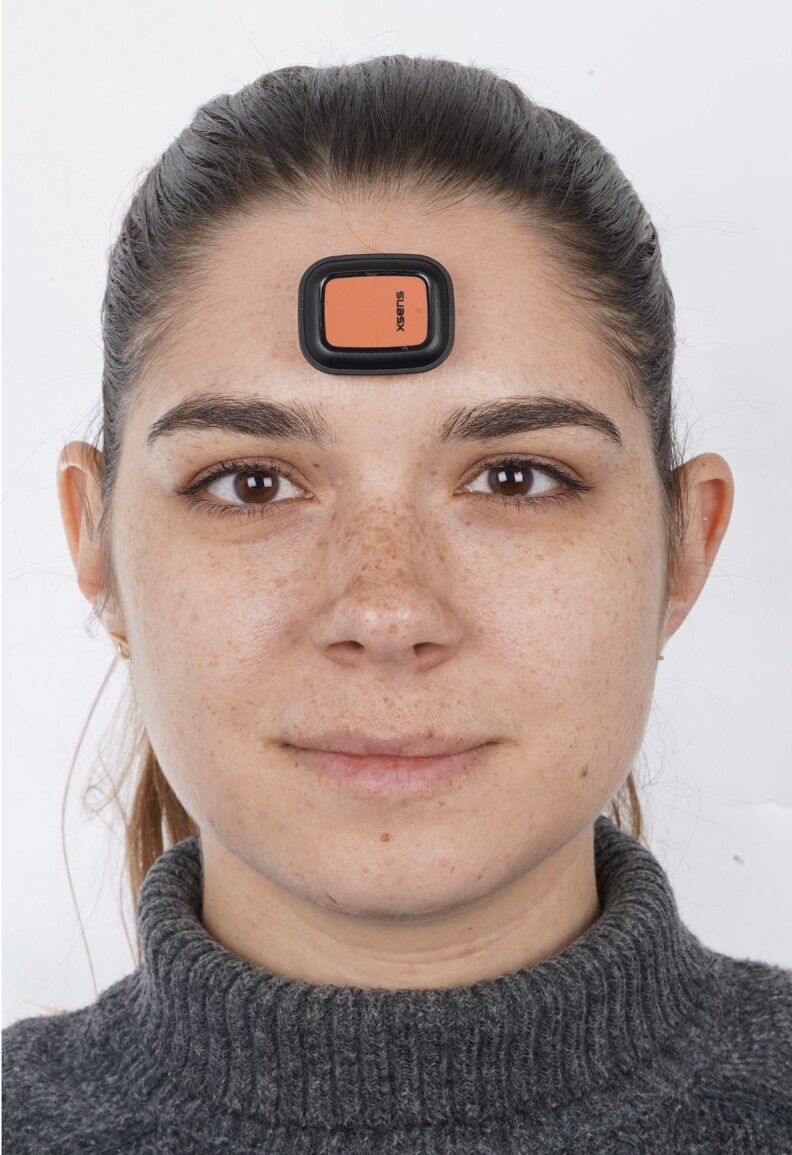
Head posture was recorded using a triaxial IMU - inertial measurement unit (SENS motion®, SENS Innovation ApS, Denmark) capable of capturing real-time three-dimensional orientation data (pitch, roll, and yaw angles). The IMU was calibrated on a horizontal surface prior to each recording to define a neutral position (0° for each axis). The sensor was affixed to the participant’s forehead using a hypoallergenic double-sided adhesive tape, ensuring it was positioned parallel to the bipupillary line. This placement allows accurate registration of head orientation while minimizing measurement errors associated with sensor misalignment, as previously validated in earlier methodological studies (Al-Yassary et al., Sci Rep 2021 [18]; Al-Yassary et al., J Oral Rehabil 2022 [17]). Participants were seated in a standardized 90–90–90 posture (hips, knees, and ankles flexed at 90°) and instructed to look at their own eyes in a mirror for 30 seconds to establish a mirror-guided natural head posture. The mirror was then covered, and participants maintained their self-balanced head posture for 5 minutes without visual feedback. Throughout this period, the IMU continuously recorded angular displacements of the head in three axes: pitch (anteroposterior inclination; flexion/extension), roll (lateral tilting; right/left inclination), and yaw (horizontal rotation; right/left turning). The collected data were subsequently averaged over the 5-minute recording period to provide a stable representation of each participant’s natural head posture.

**Figure 2.**
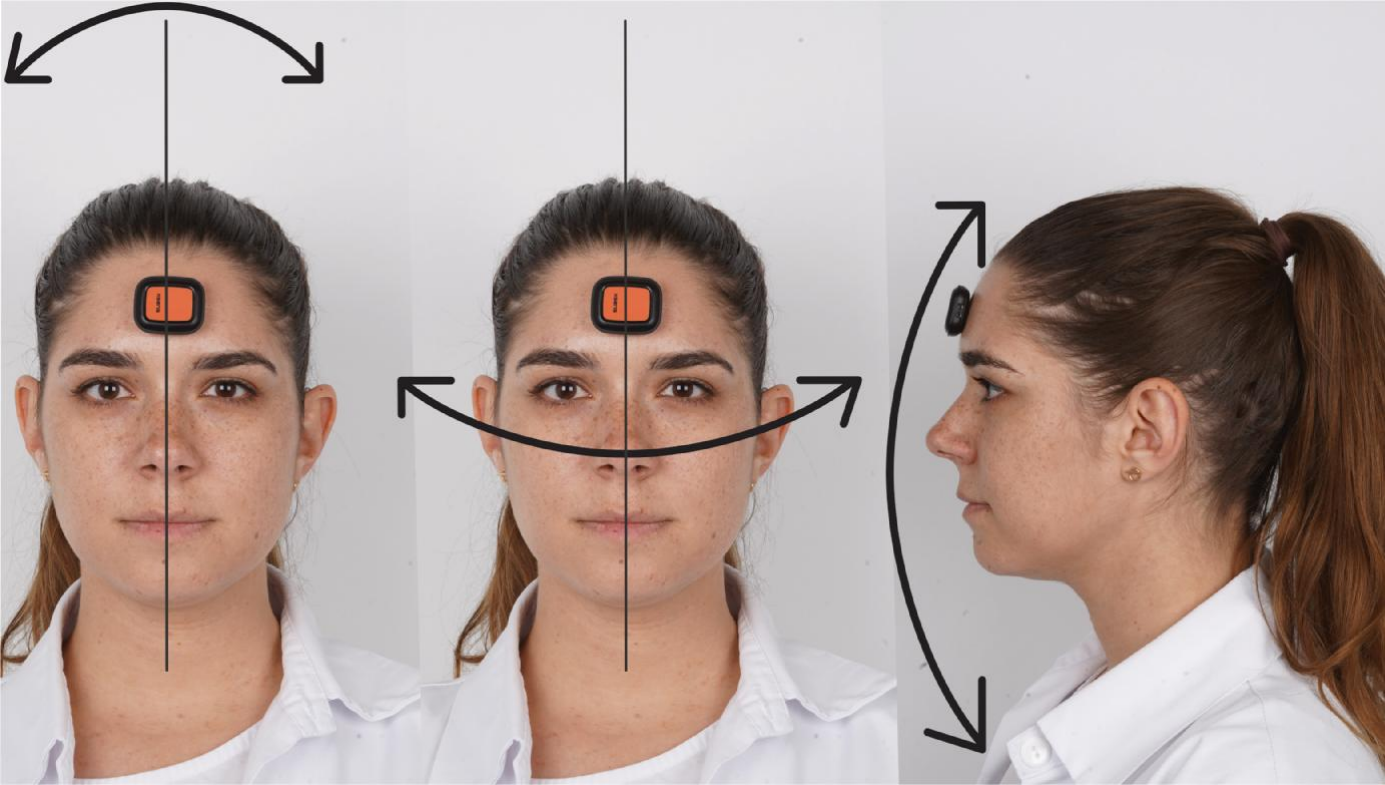
Orientation of the head in terms of pitch, roll, and yaw movements describing the three degrees-of-freedom of a human head. The left image shows roll changes, the centre image yaw changes, and the right image pitch changes.

Throughout this experimental procedure, an IMU was used to record head position continuously, which was calibrated on the ground to establish a neutral position (0° for each axis) (Fig. 1). After appropriate calibration, the IMU was placed on the participant’s forehead, parallel to the bipupillary line to minimize measurement errors related to sensor placement. The device records pitch, roll, and yaw angles to capture head movements accurately over the 5-minute period (Fig. 2) [17].

In addition to the head posture assessment, the dominant eye was determined using the aiming test described by Battista Della Porta in 1593. Each participant was instructed to point at a distant object while keeping both eyes open. They then alternately closed each eye to identify which one maintained alignment with the object. This eye was recorded as the dominant one. This procedure was carried out for all participants during their initial session (T0).

### Statistical analysis

Head posture was measured as the average over the 5 minutes of analysis across the three axes. The mean values and standard deviations were calculated for the 5-minute analysis providing a comprehensive understanding of head posture dynamics throughout the study period. An intention-to-treat analysis was planned in the case of dropouts.

Since data concerning the roll and yaw axes of head posture are sided (towards the left or right), the data were adjusted so as to normalize all data independent of sidedness. This convention simulates all crossbites as right sided, allowing us to detect differences between the control and crossbite groups without bias from the crossbite side.

SPSS [20] was used for the statistical analyses. At T0 (before treatment), comparisons between the crossbite and control groups were made using independent-sample *t*-tests to evaluate initial head posture differences. After the retention phase (T1), similar comparisons between the two groups were conducted using independent-sample *t*-tests to assess post-treatment difference in NHP between the groups.

For longitudinal changes within each group (comparing T0 to T1 within each group), paired *t*-tests were used to analyse changes separately for both the crossbite and control groups. Additionally, relevant comparisons between left- and right-sided crossbites were made using an independent-sample *t*-test, to evaluate whether the side of the crossbite had an impact on head posture outcomes.

The method error was assessed by evaluating head posture variation across duplicate recordings from 20 participants in a previous study [17] and calculating intraclass correlation coefficients (ICCs). Reliability was moderate to excellent (ICCs: 0.74–0.96), with the roll axis showing the best reliability, followed by yaw and pitch. No significant systematic differences were observed (*P* > .05), indicating good reproducibility and typical biologic variation in head posture.

Given that multiple comparisons were carried out across three axes (pitch, roll, and yaw), a Bonferroni correction was applied to adjust for potential Type I errors. Given that three comparisons (pitch, roll, and yaw) were made on two different sessions (T0 and T1), amounting to 12 comparisons, the significance threshold for each test was adjusted to an alpha level of .004 (=.05/12). Consequently, only *P*-values below the adjusted threshold of .004 were considered statistically significant.

## Results

### Sample

The present study sample comprised 40 participants, with 18 females and 22 males. The treatment group included 20 growing children (10 males and 10 females) who presented with unilateral functional posterior crossbite, and their average age at the start of the study (T0) was 8.9 ± 0.8 years. The control group consisted of 20 growing children (12 males and 8 females) without any transverse malocclusions, with an average initial age (at T0) of 9.6 ± 0.9 years. Within the crossbite group, 10 patients were treated with a quadhelix appliance, and 10 with a Hyrax-type expander. All cases resulted in successful correction of the crossbite and resolution of the mandibular functional shift, with results remaining stable over the study period (2.5 years after the end of expansion).

Although the control group was initially intended to remain untreated, two participants began orthodontic treatment with removable appliances towards the end of the follow-up period. One patient received a maxillary plate with spurs to address a thumb-sucking habit during sleep, while another patient was treated with a maxillary plate featuring an anterior bite plane to reduce a potentially traumatic deepbite.

All patients who met the study’s eligibility criteria agreed to participate. However, two participants in the crossbite group were lost to follow-up—one due to relocation and another due to medical reasons—while one participant in the control group was also lost due to relocation. The two patients in the control group who started treatment did so just before the final follow-up (T1), and their data at T0 and T1 were included in the analysis. An intention-to-treat approach was applied to analyse all initially included participants.

### Difference in head posture between children with or without unilateral functional posterior crossbite

Before treatment (T0), no statistically significant differences were observed between the crossbite and control groups with regard to head posture. In the post-retention recordings (T1), 2.5 years following successful correction of the unilateral functional posterior crossbite, no statistically significant differences were found for the three head posture axes between the groups, although for the pitch axis, there was a tendency for a higher angle in the treated crossbite subjects (*P* = .008, considered non-significant when applying the Bonferroni correction) (Table 1).

**Table 1.** Pre- and post-treatment head posture analysis.

	Crossbite group		Control group		
	mean ± SD	CI 95%	mean	CI 95%	*P*-value
**Pitch**					
T0	6.6° ± 5.8°	3.8; 9.5	4.6° ± 7.8°	0.8; 8.5	.38
T1	12.2° ± 6.2°	9.1; 15.2	5.2°± 8.6°	0.9; 9.4	.008
**Roll**					
T0	−0.5° ± 2.8°	−1.9; 0.8	−0.6° ± 5.1°	−1.9; 0.8	.94
T1	0.6° ± 2.9°	−0.9; 2.0	0.01°± 3.6°	−1.8; 1.8	.61
**Yaw**					
T0	0.5° ± 4.5°	−1.8; 2.7	0.5° ± 4.5°	−0.7; 2.7	.46
T1	1.4° ± 7.5°	−2.3; 5.2	0.7°± 3.7°	−1.2; 2.5	.71

### Changes in head posture during growth in untreated children without crossbite

In the control group, no significant differences were observed between the two sessions in the control group, indicating that no statistically significant changes occurred in head posture over time in this group (Table 2).

**Table 2.** Head posture comparison between the two time points in the control group.

	Pitch (°)		Roll (°)		Yaw (°)	
	mean ± SD	CI 95%	mean ± SD	CI 95%	mean ± SD	CI 95%
T0	4.6° ± 7.8°	0.8; 8.5	−0.6° ± 5.1°	−1.5; 1.9	1.6° ± 6.9°	−0.7; 3.8
T1	5.2° ± 8.6°	0.9; 9.4	0.01° ± 3.6°	−1.6; 2.9	0.7° ± 3.7°	−3.6; 1.7
*P*-value	.83		.55		.43	

### Changes in head posture following the correction of unilateral functional posterior crossbite

Statistically significant differences were not found following treatment of the unilateral functional posterior crossbite, although for the pitch axis (Table 3), there was a tendency for this to increase after crossbite correction (although not significantly when applying the Bonferroni correction).

**Table 3.** Head posture comparison between the two time points in the crossbite group.

	Pitch (°)		Roll (°)		Yaw (°)	
	mean ± SD	CI 95%	mean ± SD	CI 95%	mean ± SD	CI 95%
T0	6.6° ± 5.8°	3.8; 9.5	−0.5° ± 2.8°	−1.9; 0.8	0.5° ± 4.5°	−1.8; 2.7
T1	12.2° ± 6.2°	9.1; 15.2	0.6° ± 2.9°	−0.9; 2.0	1.4° ± 5.5°	−2.3; 5.2
*P*-value	.007		.16		.69	

### The influence of crossbite sidedness

When comparing absolute head posture in patients with a left-sided posterior crossbite versus those with a right-sided posterior crossbite, no statistically significant differences were found in any of the head posture axes either prior to (T0) or after (T1) crossbite correction. This indicates that the side of the crossbite does not seem to influence head posture.

### Eye dominance

26 participants had right-eye dominance whereas 12 had left-eye dominance. A comparison was carried out between these two groups with regard to head posture in each of the three axes. A statistically significant difference was found for the yaw axis between the right- and left-eye-dominant groups. Participants with right-eye dominance exhibited a mean yaw angle of 3.61° ± 3.46°, compared to −1.47° ± 3.46° in the left-eye-dominant participants (*P* < .001) (Table 4). No significant differences were observed for the pitch or roll axes (*P* = .86 and *P* = .32, respectively). These findings suggest that eye dominance may influence the orientation of head posture along the yaw axis.

**Table 4.** Head posture comparison between left- and right-eye dominant group.

	Pitch (°) mean ± SD	Roll (°) mean ± SD	Yaw (°) mean ± SD
Left dominant eye	5.10° ± 6.24°	−1.37° ± 3.64°	−1.47° ± 3.48°
Right dominant eye	5.55° ± 7.49°	0.08° ± 4.25°	3.61° ± 3.46°
*P*-value	.86	.32	<.001

## Discussion

The present prospective controlled clinical trial suggests that, in a sample of 8- to 11-year-old children, the presence of a unilateral functional posterior crossbite does not appear to influence head posture, when compared with a control group without crossbite. Moreover, successful unilateral functional posterior crossbite correction does not seem to alter head posture. At baseline, the crossbite cohort showed mean pitch, roll, and yaw angles of 6.6° ± 5.8°, −0.5° ± 2.8° and 0.5° ± 4.5°, respectively, virtually identical to controls (4.6° ± 7.8°, −0.6° ± 5.1°, 0.5° ± 4.5°; all *P* ≥ .38). Thirty months after quadhelix or Hyrax expansion (including 9 months of passive retention and the elimination of the functional shift), head posture again matched controls: pitch 12.2° ± 6.2° vs 5.2° ± 8.6°, roll 0.6° ± 2.9° vs 0.0° ± 3.6°, and yaw 1.4° ± 5.5° vs 0.7° ± 3.7°. Within-group analysis confirmed no substantive pre- to post-treatment change except for a borderline posterior head movement in the pitch axis (*P* = .007), supporting both null hypotheses and suggesting that posterior functional crossbite is clinically negligible in determining head posture.

Conversely, ocular dominance proved influential. Right-eye-dominant participants rotated the head 3.61° ± 3.46° towards the dominant side, whereas left-eye-dominant children showed a −1.47° ± 3.48° rotation (*P* < .001). This association between eye dominance and horizontal head rotation is consistent with previous findings in the literature, particularly the study by Pradham *et al*., which demonstrated that head and mandibular orientation are related to the dominant eye. These results confirm that ocular dominance can influence horizontal head posture as part of the oculomotor coordination aimed at optimizing binocular alignment. Our findings therefore reinforce existing evidence and indicate that the present measurement system is capable of detecting subtle but physiologically relevant variations in head orientation [21].

Despite clear evidence that unilateral posterior crossbite produces side-specific masseter muscle asymmetry on ultrasound and electromyographic recordings [1], our data indicate that this occlusal imbalance is too small a biomechanical stimulus to displace NHP. More powerful, highly variable influences appear to mask any crossbite-related signal. Mouth-breathing secondary to upper-airway obstruction may induce craniocervical extension in growing subjects [22] although not supported by all available evidence [23], and seasonal allergic rhinitis can accentuate forward-head compensations through intermittent nasal blockage [24]. Sedentary behaviours, particularly prolonged smartphone viewing, promote sustained neck flexion and increased cervical muscle load in schoolchildren [25], while both physical and mental fatigue degrade postural control via altered cortical recruitment patterns [26]. Finally, ocular dominance steers horizontal head orientation to optimize binocular sighting, as shown by the preferential yaw towards the non-dominant eye observed here and in oculomotor studies [27]. Together, these factors exert a greater biomechanical leverage than the maxillomandibular width discrepancy, rendering the postural impact of a functional posterior crossbite clinically negligible in the NHP measurement.

The present findings cannot exclude the reverse scenario whereby aberrant head posture contributes to crossbite development, especially severe deviations and of long duration. The severity of malocclusion has been suggested to be associated with incorrect frontal body posture [28]. Posterior crossbite is multifactorial: non-nutritive sucking habits are strongly associated with posterior crossbite prevalence [29]; persistent mouth breathing constricts the maxilla and promotes functional shifts [30]; skeletal pattern and genetics add further variance, yet twin analyses suggest low heritability and high environmental dependency for this trait [31]. The effect of aberrant head posture on the presence of posterior functional crossbite is still unclear but seems probably modest relative to these dominant aetiologies and would require substantially larger posture-defined cohorts to detect. A rigorous way to test this hypothesis would be a prospective study in which the index group is defined by an abnormal craniocervical posture at baseline and the primary outcome is the prevalence of posterior crossbite compared with a posture-matched control group.

When comparing patients with left- and right-sided unilateral functional posterior crossbite, no statistically significant differences were observed, regardless of the head posture axis analysed. This suggests that, in our sample and using our methodology, the sidedness of the crossbite does not appear to influence head posture in healthy patients. Consequently, orthodontic treatment for unilateral posterior crossbite does not seem to have a significant impact on head posture in this context.

The study’s limitations include the small sample size, which may limit the generalizability of the results. Moreover, the sample size calculation was based on an outcome other than head posture, and thus, the present study may be underpowered to show real differences. Future research should aim to control or take into account external variables that could affect head posture and include larger, more diverse samples to further explore the potential connections between crossbite and head posture, as well as investigate the effects in different body postures, such as standing, which may provide further insights into postural adaptations.

Furthermore, regarding the sample size, another limitation relates to the control group, in which two participants received minor orthodontic interventions near the end of the follow-up period. One participant was treated with a removable plate with spurs to stop a thumb-sucking habit, and another with a removable appliance featuring an anterior bite plane to manage a deep bite. Although these interventions were initiated after most of the observation period and are unlikely to have influenced head posture, they should nonetheless be acknowledged as a potential limitation.

In addition, another potential limitation concerns the use of two different expansion appliances within the intervention group (50% of treated patients received a Hyrax while 50% of treated patients received a quadhelix). Although both appliances followed comparable activation and retention protocols and achieved the same transverse correction, the allocation was not randomized, as the objective of the present study was not to compare appliance types but rather to evaluate head posture after successful correction of the crossbite and elimination of the functional shift between intercuspal and centric relation positions. Nevertheless, the absence of randomization could be regarded as a potential source of bias and should be acknowledged when interpreting the results.

With regard to the statistical limitations, a relevant limitation lies in the use of Bonferroni adjustments for multiple comparisons. While these adjustments are often used in the literature and aim to reduce Type I errors, they come with significant drawbacks, such as increasing the likelihood of Type II errors, thereby potentially missing meaningful findings [32].

## Conclusion

The results of the present prospective controlled clinical trial show that unilateral functional posterior crossbite does not influence head posture in growing children. Furthermore, the correction (through orthodontic treatment) of a unilateral functional posterior crossbite in growing children does not change head posture.

The study did identify however a significant influence of eye dominance on yaw orientation within head posture, with right-eye-dominant participants exhibiting greater yaw angles. This highlights the interplay between visual dominance and head posture.

## Data Availability

The data underlying this article will be shared on reasonable request to the corresponding author.
